# The Effects of Early Enteral and Parental Nutrition on Retinopathy of Prematurity: A Systematic Review

**DOI:** 10.7759/cureus.49029

**Published:** 2023-11-18

**Authors:** Vinod Kumar Mandala, Ashok Kumar Urakurva, Siddhartha Gangadhari, Rakesh Kotha

**Affiliations:** 1 Pediatrics, Kakatiya Medical College, Warangal, IND; 2 Pediatrics, Government Medical College Vikarabad, Vikarabad, IND; 3 Pediatrics, Niloufer Hospital, Hyderabad, IND; 4 Neonatology, Osmania Medical College, Hyderabad, IND

**Keywords:** preterm lbw, tpn:total parental nutrition, retinopathy of prematurity (rop), "neonate", early nutrition

## Abstract

The management of preterm newborns must consider the severe problem of retinopathy of prematurity (ROP). A systematic review has been conducted to effectively acknowledge how enteral and parenteral early nutrition affect the growth and progression of ROP. The study summarizes recent findings from various sources to give insight into the relationship between dietary practices and ROP risks. When untreated, retinopathy of prematurity (ROP) may cause severe vision loss or blindness in premature newborns. The latter two phases of ROP progression are the most serious. A child's early nutrition, both orally and intravenously, significantly impacts the severity and progression of ROP. This systematic review aims to examine the evidence linking early nutrition to ROP in premature infants. The study used Embase, Scopus, and PubMed to conduct our search. ROP, premature newborns, and nutrition were keywords used to find relevant papers. Nine research studies made it through the screening process and offered important information on the impact of diet on ROP. These studies support the idea that poor nutrition is a driving force behind the onset of ROP. The risk of ROP has been associated with postnatal development, hyperglycemia, polyunsaturated fatty acid levels, and the presence of breast milk. The outlook for ROP has also been discovered to be affected by the length of time the patient has received parenteral feeding. The incidence and severity of ROP may be mitigated by providing better nutrition to premature newborns. This comprehensive study concludes that early nutrition, both enteral and parenteral, substantially influences the development and progression of ROP in premature newborns. The significance of nutrition in newborn care is highlighted by the possibility that improved dietary methods might aid in preventing and treating this vision-threatening illness.

## Introduction and background

Retinopathy of prematurity (ROP) is an eye condition that primarily affects premature infants and can potentially cause blindness. It occurs from aberrant blood vessel formation in the retina and, if not taken seriously, often ends in severe vision impairment or even blindness. ROP undergoes five different stages. Considering the role of nutrition in this condition, doctors are expected to monitor how severe the ROP is. Having the idea that the stages range from mild (1) to severe (5), both stages 1 and 2 babies falling under that category get better without treatment and continue experiencing good vision. Healthcare professionals will keep monitoring them to confirm if the condition worsens. According to stage 3 of the ROP, babies may get better without any treatment, and some may need medication to prevent the unusual blood vessels from destroying the retina, hence causing retinal detachments [1].

Similarly, stage 4 of the ROP reveals that its partially detached retinas require treatment. Concerning the last stage, their retinas are entirely detached. In this case, babies will likely lose their vision even without medication. Stages 4 and 5 are the most critical conditions of ROP; hence, the reason behind looking for medication and early treatment is to minimize the likelihood of causing a severe problem [2].

With these ideas, parenteral nutrition is frequently administered during the neonatal period since very preterm infants always need more nutrition than can be given through enteral feeding during the weeks of the postnatal period. Ideally, parenteral feeding moment has been determined as a risk factor for ROP [3]. The retinopathy of prematurity must be navigated since current treatment techniques focus on late-stage infections. Poor general growth after extremely preterm births is a widespread issue connected to an elevated risk of retinopathy. Moreover, the loss of maternal connection results in the loss of extra elements provided in gestations on top of nutrition. Studies have shown the significance of nutrition and elements such as insulin, growth factors, and long-chain fatty acids for healthy retinal vascularization. The evidence supporting these implications and discoveries concerning newborns is growing. Even though improvements in neonatal care have increased the survival rate among preterm infants, the prevalence of ROP remains a significant cause for concern (VanderVeen et al., 2013). [4]. It is noted that various factors, such as oxygen therapy and low birth weight, impact the development of ROP. It is currently unclear how early nutrition, both enteral (through the gastrointestinal system) and parenteral (through intravenous routes), affects the development of ROP. That is the reason we want to conduct a review.

## Review

Objective

The comprehensive review aims to investigate the relationship between early nutrition, including enteral and parenteral nutrition content, timing, and way of administration, and the emergence and progression of ROP in preterm children.

Methods

The methods to be discussed here are based on a search strategy. In this case, systematic searches will be undertaken in major academic databases covering PubMed, Embase, and Scopus. Some of the keywords to be incorporated in the search terms will be a combination of keywords like "retinopathy or prematurity,” "preterm infants,” "nutrition,” "enteral nutrition," and "parenteral nutrition." Ideally, literature on the role of early nutrition (enteral and parental) on prematurity retinopathy was searched systematically, retrieved, and assessed to gather essential information and statistics that tackle nutrition's role in prematurity retinopathy. When conducting any systematic review, focusing on the literature search is crucial. The search strategy looks into sources and articles that discuss the research topic and research question to be addressed. In this context, the literature search helped determine valid and evidence-based articles that gave clear findings on the role of early nutrition in early nutrition (enteral and parental) and the role of retinopathy in prematurity. Information concerning this topic was retrieved from databases that cover journals, articles, and report papers in the medical sector. The search process followed the PICO (population, intervention, comparison, and outcome) approach as a guide, leading to the most appropriate articles or literature addressing the research topic (Table 1). The study was assigned the PROSPERO registration number 468704.

**Table 1:** PICO Framework PICO: Population, intervention, comparison, and outcome

PICO Framework	
Population	Neonates
Intervention	Early nutrition
Comparison	standard nutrition
Outcome	Retinopathy of prematurity (ROP)
Study	Observational and Randomization studies

Identification of the study

The EndNote tool was used to find all these sources for suitable articles relevant to my topic. The acquired articles were manually checked to eliminate duplicates. Choosing pertinent publications that dealt with the issue of the role of early nutrition in ROP was another step in the screening procedure. The content of the publication's topics, abstracts, crucial results, conclusions, and entire information was explored to identify which ones were effective. Various possible publications (520) with connections to the research topic were realized through the search strategy. Of the many articles found, only nine were qualified to meet the threshold to be part of the systematic literature review. Articles found by peer-reviewed articles initially determined were adopted and considered to be used in the systematic review of the literature.

Data extraction

The chosen research will obtain information relevant to our study topic, integrating study designs, sample size, nutritional interventions, and ROP incidence, severity, and significance results. To be sure of the kind of articles to be included in the systematic review, available research followed the inclusion criteria as indicated below:

Inclusion Criteria

The kind of research to be incorporated into the review has to meet different criteria. Only peer-reviewed sources published in English will be incorporated. Also, research concerning preterm infants (gestational age below 37 weeks) will be considered. Similarly, research that analyzes the influence of early nutrition (enteral and parenteral) on ROP development and severity will be valued. Inclusively, observational research incorporating cohort and case controls and randomized controlled trials will be considered.

Results

**Figure 1:**
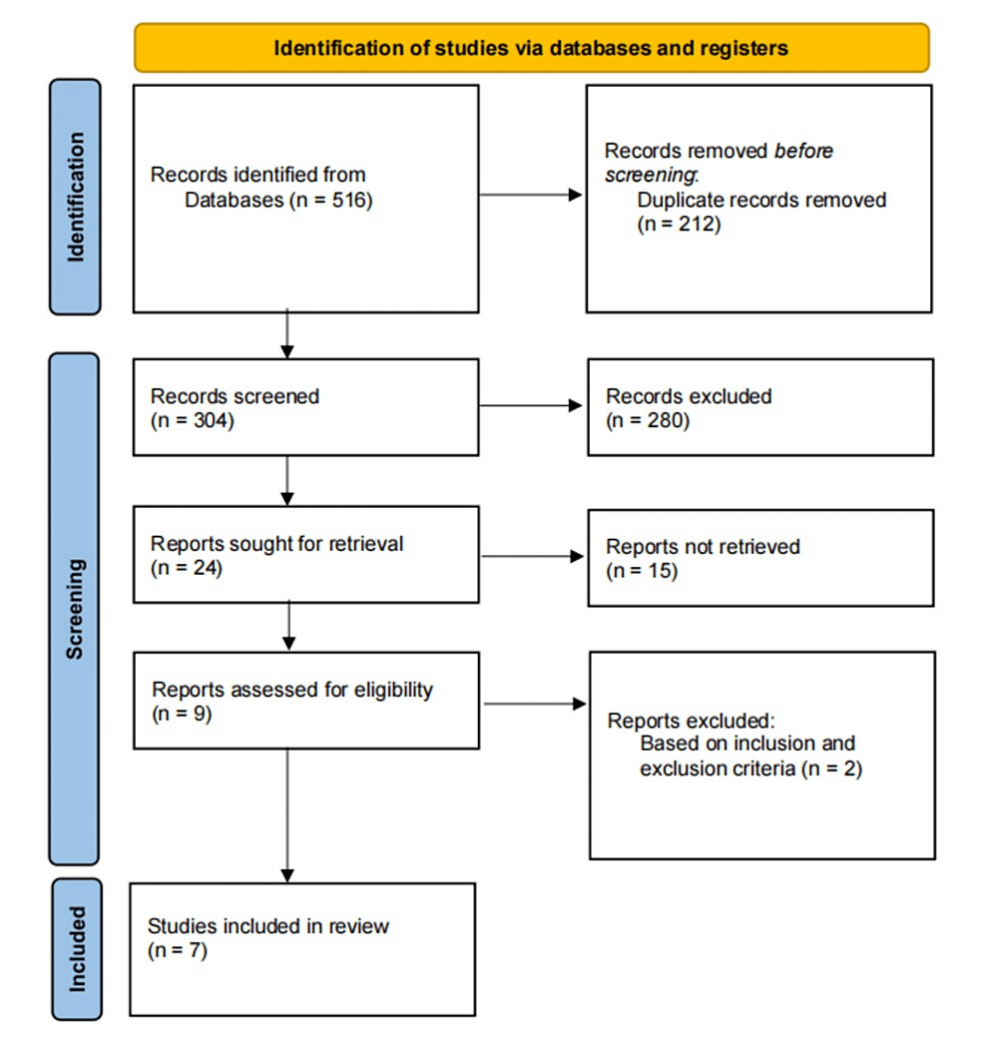
PRISMA Diagram of Article Selection

According to the first trial, when looking for the most appropriate articles suitable for the research topic, approximately 516 articles were found. After reviewing, 212 sources seemed duplicated, so they were eliminated, leaving 304 sources for further review. In addition, the review continued and found that 280 articles were irrelevant to our research. A reminder of 24 sources was noted, and then filtration was done to remain with only articles addressing issues on early nutrition's role in the ROP. After keenly considering the sources that remained with nine articles, two were disregarded as per inclusion criteria, and hence, only seven were confirmed as the outcome of the selection method (Figure 1).

Quality assessment

The Newcastle-Ottawa Scale (NOS) for observational research and the Cochrane Risks of Bias tool for randomized controlled studies (RCTs) (RoB 2) were incorporated to assess the type of research chosen to confirm the sources' validity. The chosen references were awarded a maximum of three stars. Below is a table showing incorporated sources according to the NOS scale. Because not all of these studies were observational, RoB 2 was applied to one randomized trial. This study came under low-risk bias as per the RoB-2 tool. Each article's study design and research goals are unique; thus, quality evaluation techniques should be used wisely (Table 2). 

**Table 2:** Quality Ratings of the Selected Articles Based on the Newcastle-Ottawa Scale Total Score with * Unsatisfactory studies *** Satisfactory studies **** Good studies ***** Very good studies

SN	Study	Selection	Comparability	Outcomes
1	Zhou et al. (2021)[6]	***	**	***
2	Pivodic et al. (2023) [7]	***	**	***
3	Kumar et al. (20200) [8]	***	**	***
4	VanderVeen et al., (2013) [4]	***	**	***
5	Klevebro et al., (2019) [9]	***	**	***
6	Porcelli PJ et al. (2010)[10]	***	**	***

Description of studies

In accordance to the PRISMA framework, seven studies were included in this review (Table 3). Zhou et al. (2021) explored nutrition's role in prematurity retinopathy [2]. These authors shed light on the fact that retinal prematurity is one of the core causes of blindness among infants during their preterm. Based on the ROP, the study argued that perinatal factors such as oxygen and inflammation affect retinal blood vessel formation, which is prematurely interfered within preterm newborns. The risk of ROP would have been reduced by improving nutritional activities for preterm infants. The evidence sustaining the possible effects of postnatal growth, hyperglycemia, polyunsaturated fatty acids, and break milk on ROP risks was reviewed in this article. Similarly, the study explained the vision outcomes for children influenced by ROP and the temporary management approaches for the condition. Regarding the research question and topic, the study concluded that nutrition played a crucial role in retinopathy of prematurity.

**Table 3:** Summary of Selected Article AGA: Appropriate for gestational age; BPD: Bronchopulmonary dysplasia; ROP: Retinopathy of prematurity.

S. No.	Authors	Study Type	Country	Gestation Age	Condition/Outcome
1	Zhou et al. (2021) [2]	Observational Study	China	Preterm neonates	Improving nutritional practices for preterm infants may lessen the risk of ROP
2	Pivodic et al. (2023) [7]	Observational Study	Sweden	Newborn infant (24 to <31 weeks)	Treatment of retinopathy of prematurity
3	Kumar et al. (2020) [7]	Observational Study	India	Preterm neonates (<34 wks, <1800grams)	Reducing sight threatening ROP
4	VanderVeen et al., (2013) [4]	Observational Study	United States	Preterm newborn (below 28 weeks GA)	Weight gain in preterm newborns and risks of ROP
5	Klevebro et al., (2019) [9]	Observational Study	Sweden	Newborn infants (Before 27 0/7 weeks)	Nutritional intakes on growth and risks of BPD and ROP
6	Can et al., (2019) [5]	Randomized controlled trial	Iran	Preterm infants (below 32 weeks GA)	Testing for insulin-like growth factor
7	Porcelli PJ, et al. (2011) [10]	Retrospective observational study	United states	AGA infants 700-1000gms	ROP surgery rates

Pivodic et al. (2023) conducted another study on DIGIDROP projection models for severe ROP [3]. According to this study, it was noted that ROP is a potentially blinding, preventable eye condition that is usually figured out in every preterm infant. Ideally, the study demonstrated that, among the significant risk variables, gestational age and weight during birth were particularly notable. Routine ROP tests were done to identify a small percentage of infants who ultimately continued to need medication. Approximately 30% of infants within Sweden who were examined are noted to have ROP, and 6% of those need medication. A safe ROP projection model was found to improve infant welfare and increase screening effectiveness by determining infants prone to low and high risks. Furthermore, the thesis's main goals were to create and confirm the project models for severe ROP that needed treatment and offer clinical advice to safely and successfully release low-risk infants from ROP screening exams. The study also illustrated the ROP's natural history, which revealed that parenteral nutrition duration (PND) on the ROP affects prognosis.

The Swedish National ROP Register offered the information incorporated here. In this thesis paper, IV included 11178 babies, and this paper demonstrated that days on parenteral nutrition are a strong predictor of any ROP and severe ROP requiring treatment. The model screening was created using logistic regression, which embraced status and age while diagnosing ROP and the DIGIROP-birth risk estimates. Conclusively, boys and girls were confirmed to have different ROP risks during GA and PND [11].

Hard, Smith, and Hellstrom (2014) researched nutrition's role in ROP [12]. These authors argued that it would be essential if retinopathy of prematurity could be avoided due to the current treatment approaches in the late stages of the disease. According to the research, it was noted that there is a relationship between nutrition and ROP; hence, the study was suitable for this research topic.

In addition, Kumar et al. (2020) did research to examine the development of quality improvements for minimizing blindness-threatening retinopathy in prematurity. Retinopathy of prematurity is becoming a leading cause of infants' blindness as preterm newborns survive [8]. The study found that the prevalence of sight conditions can be decreased by improving the care offered to premature newborns who survive longer. It was confirmed that the prevalence of ROP could be reduced by improving the care offered to premature infants. Conclusive: The research confirmed that the causality of ROP was due to poor nutrition, exposure to blood products, and unneeded oxygen administration. Therefore, it was advantageous to have chosen the source because it confirmed the importance of nutrition in ROP.

Hussein et al. (2022) confirmed that the kind of nutrition transferred from the mother to the baby helped protect the infant's immunity [13]. However, as per the study, the milk's nutrients needed to be improved for infants. The authors argued that vitamins are crucial and expected to be part of the nutrition directed to the infant to protect it from some morbidities caused by issues like retinopathy. The study confirmed that human milk provided nutrients for preterm infants. The study suits the relevancy of the research topic in this case; hence, it was worth the systematic review.

Tsang et al. (2021) confirmed that nutraceuticals affect ROP. The research noted that ROP is one of the causes of infant blindness, which is attributed to retinal neovascularization. Although treatment for ROP was found to be effective in minimizing the severity of ROP, such therapies are likely to cause long-term effects such as systemic structural complications. However, the study confirmed that nutritional supplements are crucial for newborns to recover energy supplies in their bodies. Therefore, the study concluded that nutraceuticals protect infants from retinal vasculature; hence, they could prevent and manage ROP in infants. These findings demonstrate how the research fits into the systemic review of this research topic [14].

Moreover, Vanderveen et al. (2012) examined the role of early nutrition and weight gain in preterm newborns and their risks for ROP [7]. Similarly, Klevebro et al. (2019) contributed by examining the influence of protein intakes connected to growth and ROP in preterm infants [8]. In addition, Can et al. (2013) conducted another study on insulin-like growth factors and how they could be used to prevent ROP [10]. These studies aimed to confirm any relationship between early nutrition and ROP. The findings confirmed that the studies were worth our research since they aligned with our research question and topic, that early nutrition plays a pivotal role in ROP.

Porcelli PJ, et al. reported that increasing human milk feeding rates and vitamin dosing options improved ROP surgery rates. ROP-operated infants received more parenteral nutrition and less breast milk and vitamin E in the second postnatal week. Human milk, with an odds ratio of 0.94, was a poor predictor of ROP surgery [10].

Discussion

Neonatal retinal vascularization produces vascular endothelial growth factor (VEGF), which is controlled by insulin-like growth factor 1 (IGF-1). IGF-1 aids in the survival and proliferation of newly formed endothelial cells. Insulin-like growth factor 1 (IGF-1) levels are high during fetal life due to the glycolysis of nutrients derived from the mother. Premature babies have a sudden drop in IGF-1 levels after birth. Newborns have limited physiological reserves for the formation of IGF-1. A newborn weighing less than 1,000 g typically has 1% fat. However, the retina's rapid proliferation necessitates more nutrition [15].

The findings from the given studies confirmed that ROP is one of the leading causes of infant blindness. It was noted that a lack of appropriate nutrition for infants and exposure to blood products facilitate the condition. Results show a need to transfer enough nutrition from the mother to the infant to strengthen their immunity. Ideally, nutrition is more recommendable than the ROP treatment since it could result in deformities in the systemic structure (VanderVeen et al., 2013) [4]. Therefore, early nutrition was noted to be the only solution without negative implications for preventing ROP. Thus, there is a strong correlation between early nutrition (enteral and parental) and the retinopathy of prematurity.

Consistent results from this research highlight the importance of prenatal nutrition in reducing the risk of ROP. Premature newborns, in particular, benefit greatly from receiving enough nutrition, which has been shown to lower the incidence of ROP significantly. The results of this research highlight the value of enteral and parenteral feeding in meeting the nutritional needs of preterm newborns. As the infant's immune system and general health develop, good nutrition protects against ROP. This literature review emphasizes the need for neonatal care facilities to adopt policies and procedures that place a premium on early feeding treatments.

A systematic review by Miller J. et al. reports a lower incidence of ROP in breastfed newborns [16]. Bharwani SK et al. reported in their systematic review that breast milk prevents any stage of ROP [17]. A systematic review by Vayalthrikkovil S found that parental fish oil lipid emulsions reduced the severity of ROP [18]. According to Diggikar S, polyunsaturated fatty acids did not reduce ROP incidence; however, there was a trend toward benefit in mitigating severe forms of ROP [19].

These results also imply that the treatments available for ROP may negatively affect preterm babies' overall systemic structure. Therefore, rather than depending entirely on treatment alternatives, the research advocates taking a preventative strategy through early nutrition. This view is consistent with preventive medicine, which stresses the need to get to the bottom of a problem before it spreads. These findings highlight the importance of early feeding in reducing the incidence of ROP in preterm newborns, suggesting a potentially fruitful direction for future neonatal care approaches. The systematic review here confirmed that nutrition plays a crucial role in ROP. 

## Conclusions

The goal of the systematic review was to present a comprehensive insight into the most current information concerning the impact of nutrition, including enteral and parenteral feedings, on the development and progression of ROP. Acknowledging how nutritional measures affect the retinopathy of prematurity risks can improve preterm newborn care and clinical processes, possibly reducing the prevalence and severity of this blindness illness. The studies have given different ideas, confirming a strong correlation between the role of nutrition in ROP.
